# Prospects of organic acids as safe alternative to antibiotics in broiler chickens diet

**DOI:** 10.1007/s11356-022-19241-8

**Published:** 2022-02-23

**Authors:** Rifat Ullah Khan, Shabana Naz, Fazal Raziq, Qudratullah Qudratullah, Nazir Ahmad Khan, Vito Laudadio, Vincenzo Tufarelli, Marco Ragni

**Affiliations:** 1 College of Veterinary Sciences, Faculty of Animal Husbandry and Veterinary Sciences, The University of Agriculture, Peshawar, Pakistan; 2 Department of Zoology, Government College University, Faisalabad, Pakistan; 3 Livestock and Dairy Development, Peshawar, Pakistan; 4 Department of Surgery and Pet Centre, Cholistan University of Veterinary and Animal Sciences, Bahawalpur, Pakistan; 5 Department of Animal Nutrition, Faculty of Animal Husbandry & Veterinary Sciences, The University of Agriculture, Peshawar, Pakistan; 6 Department of DETO, Section of Veterinary Science and Animal Production, University of Bari ‘Aldo Moro’, Valenzano, Bari, Italy; 7 Department of Agro-Environmental and Territorial Science, University of Bari ‘Aldo Moro’, Bari, Italy

**Keywords:** Organic acid, Broiler, Growth, Immunity, Health

## Abstract

Genetically, modern broilers are fast-growing birds which attain the market age at the age of 5 weeks. To maintain optimum production, antibiotics have been commonly included in the diets as growth promoters. However, due to the increase in antimicrobial resistance, their uses have been banned worldwide. To keep the optimum level of production and health in broiler industry, the use of alternative growth promoters such as probiotics, prebiotics, enzymes, and organic acids has been proposed. Chemically, organic acids are weak acids and only partially dissociate. They are considered safe and have been used for preservation of food for centuries. Nowadays, organic acids have been reported for antibacterial, immune potentiating, and growth promoters in broilers. In this review, the effects of dietary inclusion of organic acids on growth, nutrient digestibility, intestinal integrity, immune system, and antibacterial activity in broilers are discussed.

## Introduction

Feed additives are now considered essential for the optimum performance and productivity in modern poultry production (Shahid et al. 2015). In the past, antibiotics have been used as growth promoters for enhanced production and balancing intestinal flora (Garcia et al. 2007; Abudabos et al. 2016; Haulisah et al. 2021). Antibiotic-resistant bacteria could easily be transmitted from animals to the human through food chain (Chowdhury et al. 2009; Khan et al. 2016a). The use of subtherapeutic level of antibiotics has a viable role in enhancing poultry production for many years. However, this practice was discouraged due to the emergence of antibiotic resistance of animal and human pathogens. The European Union was the first organization which indicated its intention to withdraw the use of antibiotic in 2006. The search for an alternative was therefore suggested to provide suitable alternative role which could maintain the growth and feed efficiency in the farm animals (Scicutella et al. 2021).

**Fig. 1:**
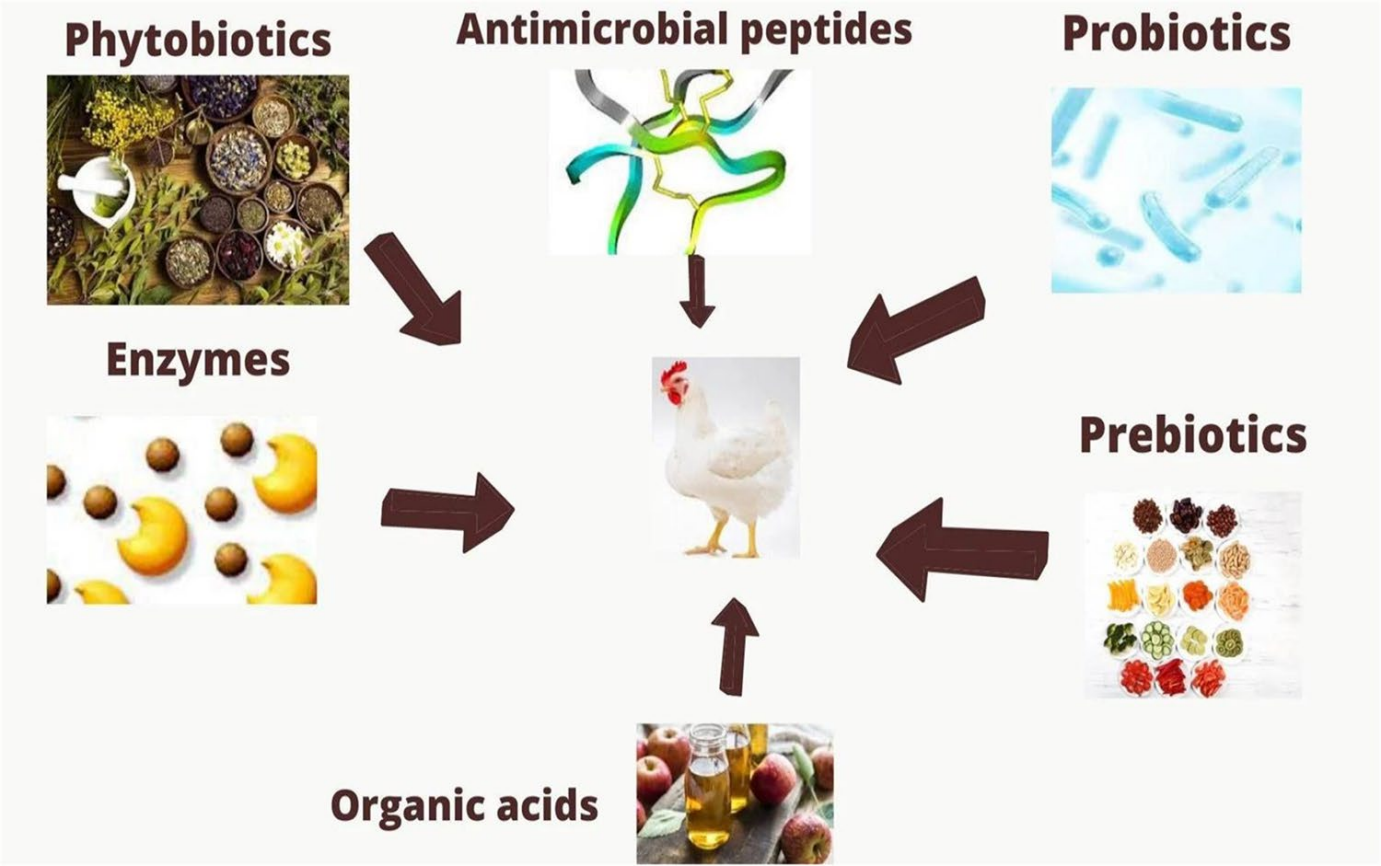
Example of some alternatives to antibiotics used in broiler production

Recently, the use of antibiotics as growth promoters has been banned due to the emerging resistance against microbes and their residues in meat and egg (Cakir et al. 2008; Dhama et al. 2015; Ullah et al. 2022). Due to the ban on the use of antibiotics in poultry production, there are compelling reasons to use other alternatives to maintain optimum poultry production (Raza et al. 2016; Chand et al. 2016; Tehseen et al. 2016; Hafeez et al. 2021). Therefore, there is a need to search for alternatives, especially in view of recent withdrawal by the European Union (Khan et al. 2012a; Alzawqari et al. 2016; Abudabos et al. 2018). Several feed additives have been proposed such as probiotic (Khan and Naz 2013; Khan et al.2013a, b, 2014a, b; Alam et al. 2020; Shah et al. 2020), prebiotics (Chand et al.^2016^, ^2019^; Tufail et al. 2019; Haq et al. 2020), enzymes (Abd El-Hack et al. 2018; Sultan et al. 2019; Alam et al. 2020; Jabbar et al. 2021a, b), and herbs (Khan et al. 2012a, b, c, d; Mushtaq et al. 2013; Dhama et al. 2014; Alzawqari et al. 2016; Hafeez et al. 2020a, b, c; Ahmad et al. 2020; Chand et al. 2021; Israr et al. 2021; Khan et al. 2021a, b; Shuaib et al. 2021a, b), which have been reported in poultry (Fig. 1) and illustrated by Gadde et al. (2017). Among these, the organic acids (propionic acid, formic acid, citric acid, acetic acid) are promising alternatives. Their inclusion in the broiler feed has been shown to enhance the feed intake, growth, and feed efficiency.

Acidifiers play a key role in the preservation of feed stuff without antibiotics. For centuries, organic acids have been used as feed preservative due to their strong antibacterial and antifungal properties (Hernandez et al. 2006). For effective livestock production, a constant supply of high-quality feed should be available all year. Even in hygienic settings, variables like high moisture and a warm atmosphere might encourage the growth of fungi, yeast, or bacteria, reducing the nutritional content of the feed by metabolizing its carbohydrates and protein. Benzoic acid, acetic acid, and their salts, such as sodium benzoate and sodium acetate, can be used as feed preservative (Pearlin et al. 2020). Organic acids or salts can be added to poultry feed at a rate of 0.5 kg/ton for mold control and 2.5–3.0 kg/ton for pH decrease and *Salmonella* control (Banupriya et al. 2016). Organic acids individually or their combinations are usually considered safe and perform similar functions like antibiotics (Adil et al. 2010). The antimicrobial mechanism of organic acid has been suggested to the lowering of pH of the gut, thereby limiting the growth of the bacteria less tolerant to acid pH (Hernandez et al. 2006). The antimicrobial activity of the organic acid is linked with either simple monocarboxylic acid such as butyric acid, propionic acid, acetic acid and formic acid, or carboxylic acid having a hydroxyl group such as tartaric acid, citric acid, malic acid, and lactic acid. Most acids having antimicrobial activity have a pKa value between 3 and 5. The molecular weight, pKa value, shape (undissociated or dissociated), and particular antibacterial action (targeted microorganism) all influence organic acid efficiency (Aljumaah et al. 2020). Chemically, organic acids are weak acids and dissociate only partially. Most of the organic acids are available as sodium, potassium, and calcium salts with varying physical and chemical characteristics. The magnitude of antimicrobial activity of an acid depends upon its concentration and pH (Chaveerach et al. 2004). Moreover, each of the acid has a specific spectrum of antimicrobial activity. For example, lactic acid is more effective against bacteria, whereas sorbic acid acts is known for its anti-mold activity (Dibner and Buttin 2002). Some other organic acids have a wide range of the spectrum. For example, formic acid and propionic acid are antibacterial and antifungal. Microencapsulation is another option, as the inert film that surrounds the organic acid particles allows for a more efficient release of these compounds (Galli et al. 2021). As a result, the therapeutic activity is spread throughout the gut. In the previous reviews, organic acids in poultry have several deficiencies. The current review provides some new aspects not covered by previous reviews. For example, Scicutella et al. (2021) reported a combination of phenolic compounds and organic acids. Similarly, other previous reviews have not covered some of the important aspects such as growth and nutrient digestibility. Important aspect of the present review is that it has described in detail the mechanism of action of organic acid producing benefits to the poultry producers.

## Classification and uses of organic acids

Organic acids are composed of an organic carboxylic acid such as amino acids and fatty acids having R-COOH structure. Only limited numbers of these acids have an effect on the gut microflora. Organic acids have long been used as additive for the reduction in food deterioration and prolonging the shelf life of food commodities especially perishable food ingredients. Organic acid is generally composed of saturated straight-chain monocarboxylic acids or their derivatives (Ricke, 2003). Some of the acids, such as butyrate, propionate, and acetate, are produced in the GIT of the animals where the anaerobic environment is predominant. Organic acids were originally added in the food animal industry as an antifungal agent; however, in the past, there is evidence that the different acids such as formic acid and propionic acids have been used for their antibacterial activities against contaminated food-borne pathogens especially *Salmonella* species (Ricke, 2003). Some of the examples of the positive effects of organic acids in broilers are given in Tables 1 and 2.

**Table 1:** Some commonly used organic acids, their chemical name, formula, pKa value, and their positive effects in broiler

Organic acid	Chemical formula	Chemical name	pKa	Physical state	Positive effects	References
Formic acid	HCOOH	Formic acid	3.75	Liquid	Physical growth, digestibility, immunity, antimicrobial	Garcia et al. (2007), Ghazala et al. (2011), Abbas et al. (2013), Koyuncu et al. (2013), Hernandez et al. (2006), Abbas et al. (2013)
Acetic acid	CH_3_COOH	Acetic acid	4.76	Liquid	Antibacterial	Juneja and Thippareddi (2004)
Citric acid	COOHCH_2_C(OH)(COOH)CH_2_COOH	2-Hydroxy-1,2,3-propanetricarboxylic acid	3.13	Solid	Antibacterial, physical growth, digestibility, immunity	Gonzalez-Fandos et al. (2020), Haque et al. (2009), Ao et al. (2009), Nourmohammadi et al. (2012), Chowdhury et al. (2009), Moghadam et al. (2006)
Butyric acid	CH_3_CH_2_CH_2_COOH	Butanoic acid	4.82	Liquid	Physical and intestinal growth, immunity	Adil et al. (2010), Leeson et al. (2005), Panda et al. (2009), Zhang et al. (2011)
Fumaric acid	COOHCH:CHCOOH	2-Butenedioic acid	3.02	Solid	Physical and intestinal growth, digestibility	Adil et al. (2010), Ghazala et al. (2011), Runho et al. (1997)
Lactic acid	CH_3_CH(OH)COOH	2-Hydroxypropanoic acid	3.83	Liquid	Physical and intestinal growth, antibacterial	Adil et al. (2010), Sultan et al. (2015)
Malic acid	COOHCH_2_CH(OH)COOH	Hydroxybutanedioic acid	3.4	Liquid	Physical growth and feed efficiency	Vogt et al. (1981)

**Table 2:** Example of type of organic acid, dose, and effect against different pathogens in broilers

Organic acid	Dose	Effects	References
Formic acid + sodium formate	0.9%	Antimicrobial effect against *Salmonella typhimurium*	Adhikari et al. (2020)
Mainly formic acid	0.5 kg/ton	Antimicrobial effect against *Campylobacter coli*	Mortada et al. (2020)
Formic + propionic acids and their salts	0.1, 0.02, 0.04%	Antimicrobial effect against *E. coli* K88	Emami et al. (2017)
Wheat bran fermented fatty acids	0.1%	Antimicrobial against *Salmonella*	Vermeulen et al. (2017)
Medium chain fatty acids	Unknown	Antimicrobial effect against *Salmonella typhimurium*	Abudabos et al. (2017)
Short and medium chain fatty acids	0.5–2.5 g/kg	Antimicrobial effect against	Kumar et al. (2021)

## Growth-promoting effect

In most of the instances, growth-promoting effects have been reported in broiler in response to organic acid supplementation. The improved growth-promoting effects of the organic acids could possibly be due to the better feed utilization, the reduction in pathogenic microbial load, and the provision of a suitable environment for the growth-enhancing bacteria (Khan et al. 2016a; Baghban-Kanani et al. 2019). Chowdhury et al. (2009) found that supplementation of 0.5% citric acid improved feed intake, growth, carcass yield, and bone ash in broiler. Abdel-Fattah et al. (2008) reported that live body weight was improved in chicks fed a diet supplemented with acetic acid, citric acid, and lactic acid. Moghadam et al. (2006) found that feed consumption in broiler improved when supplemented with citric acid. Improved feed intake and feed efficiency were also reported by Atapattu and Nelligaswatta (2005). Nezhad et al. (2007) reported that feed efficiency improved in broiler fed with citric acid. Vogt et al. (1981) found that supplementation of ascorbic, malic acid, and tartaric acid increased weight gain and feed efficiency. Panda et al. (2009) reported improved broiler weight in response to 0.4% butyrate in comparison with antibiotic dose. Similarly, improved broiler weight and feed efficiency have been reported by other researchers (Leeson et al. 2005; Adil et al. 2010; Rodjan et al. 2017; Yang et al. 2018; Araujo et al. 2019; Nguyen and Kim 2020; Sureshkumar et al. 2021). Adhikari et al. (2020) also reported improved growth performance in *Salmonella typhimurium*-infected broilers in response to supplementation of 0.9% organic acid. Sabour et al. (2019) fed a diet supplemented with a combination of sodium butyrate, citric acid, phosphoric acid, acetic acid, propionic acid, formic acid, and lactic acid and reported improved growth performance in broilers. Emami et al. (2017) found improved growth performance in broilers infected with *E. coli K88*, which was attributed to the enhanced nutrient digestibility.

The improved performance of broilers in response to the supplementation of organic acid is due to enhanced digestibility of energy and protein contents of the feed and reduction of microbial pathogens, improving the immunity, lowering the infectious level, and reducing the ammonia and other harmful metabolites (Khan et al. 2016a). The improved FCR could be probably due to the lower feed intake and better utilization of nutrients resulting in a higher weight gain in broiler supplemented with organic acids. In addition, the undissociated organic acid could easily penetrate the lipid bi-layers of the cell membrane of the molds and bacteria. Inside the cell, the proton is released by the organic acid in the presence of the alkaline environment, resulting in the reduction of the intracellular pH (Hernández et al. 2006). This process is followed by the enzymatic reaction forcing the bacterial cell to release proton and the accumulation of intracellular anion. Other beneficial impacts include the release of digestive enzymes and microbial phytase activity, pancreatic secretion, and proliferation of intestinal cells (Dibner and Buttin 2002).

The literature reports on how the dietary fermentable fiber fraction may be used to create bioactive fatty acids in the animal gut, which have an antibacterial impact, as one of the most current techniques to supplement diets with organic acids (Yudiarti et al. 2020). Wheat bran, a by-product of wheat milling, was added to feed to see how effective it was against *Salmonella* in terms of percent and particle size. The genes that control *Salmonella* pathogenicity were downregulated as a result of a quick fermentation process that generated butyric acid (Scicutella et al. 2021). In addition, organic acids have been used to improve the meat quality. In this way, a dose of 300 mg/kg glycerol monolaurate can exert positive effect on improving the nutritional quality of meat (Fortuoso et al. 2019).

## Antimicrobial effect of organic acid

In poultry production, organic acids have been used as a tool to reduce pathogenic bacteria. The important pathogenic bacteria colonizing the intestinal tract include *Escherichia coli*, *Campylobacter*, and *Salmonella*. Many of the reports have documented positive effects of organic acids on the reduction of these pathogenic bacteria in drinking water and the part of the gastrointestinal tract (GIT), ultimately leading to improved feed utilization and growth performance (Khan et al. 2016b). Koyuncu et al. (2013) found a reduction in *Salmonella* in feed treated for 5 days with 1% formic acid. Gonzalez-Fandos et al. (2020) reported that citric acid solution reduced the growth of *Listeria monocytogenes* on poultry legs stored at 4 °C for 8 days. Adhikari et al. (2020) found that addition of 0.9% organic acid (mainly sodium format) in the diet of broilers resulted in reversing the *Salmonella typhimurium* colonization in broiler chickens. Mortada et al. (2020) concluded that organic acid (formic acid, cinnamaldehyde) significantly inhibited the proliferation of *Campylobacter coli* population in the in vitro study although its effect on cecal load and carcass surface remained no significant. Kumar et al. (2021) reported that organic acid is effective in decreasing cecal *Clostridium perfringens* shedding in broilers during experimental induction of the infection. Beneficial bacteria were increased while *E. coli* infestation decreased in response to organic acid supplementation in a 35-day experiment (Emami et al. 2017). Improved beneficial bacterial profile (increased *Lactobacillus* spp*.*) was also found to be improved in response to organic acid in broilers in several studies (Rodjan et al. 2017; Nguyen and Kim 2020; Sureshkumar et al. 2021).

An important source of *Campylobacter* infection is poultry products, since horizontal and vertical transmission of *Campylobacter* are unlikely to occur. In addition, *Campylobacter* can survive in drinking water for a long time (Chaveerach et al. 2004). Chaveerach et al. (2004) reported that the signs of *Campylobacter* infection in the gut disappeared by drinking acidified water. The antibacterial activity of the organic acids is not fully understood. The literature indicated that the effects of different organic acids depend on the type of organism, chemical composition, animal species, exact location in the intestines, and buffering capacity. The positive effects of antibacterial activity of organic acid have been linked with the physical chemistry of the acid, bacterial species, media composition, and growth conditions (Ricke 2003). The use of citric acid improves the development of *Lactobacillus* spp*.* in the gut and inhibits the growth and proliferation of pathogenic bacteria such as *Salmonella* and *Escherichia coli* by activating the proteolytic enzymes, absorption of minerals, reduction of ammonia and growth-depressing microbial metabolites, and stimulation of feed intake (Chowdhury et al. 2009).

**Fig. 2:**
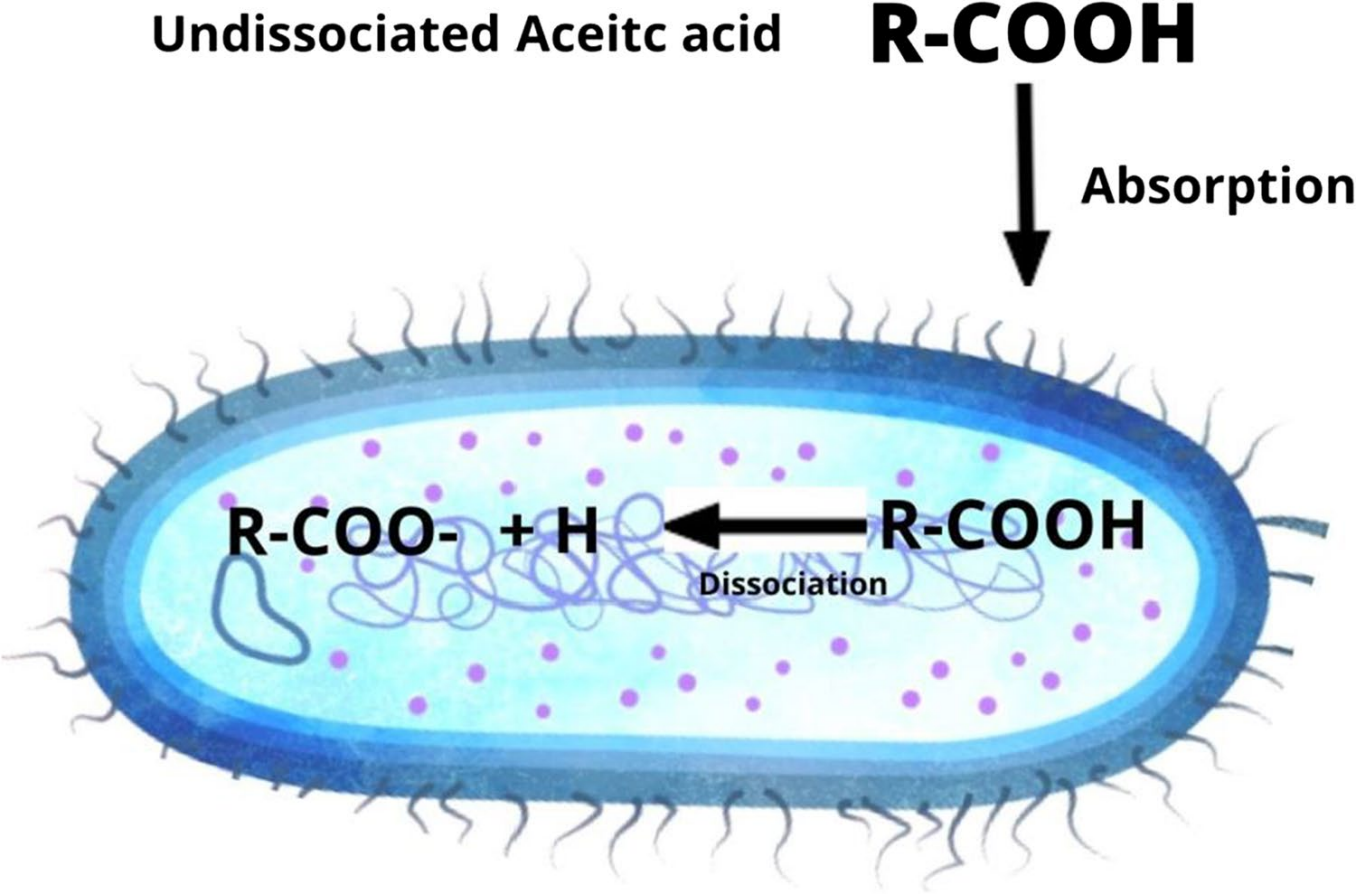
Mechanism of action of organic acid against bacteria

The mechanism of action of organic acids is quite different from that of inorganic acid. According to Dibner and Buttin (2002), the mechanism of action of organic acids is linked with its special characteristic of dissociation. Under low pH, the acid is more available in the dissociated form. The undissociated form of organic acids is more lipophilic in nature and can easily diffuse into the cell membrane of bacteria and molds (Fig. 2). The low pH inside the cell causes disruption of the enzymatic reaction and transport system (Scicutella et al. 2021). In addition, the low pH disrupts the energy generation process, which ultimately leads to prevention of the bacterial cell proliferation and growth resulting in some degree of bacteriostasis (Khan and Iqbal 2016; Scicutella et al. 2021). The direct antibacterial effect of organic acid allows the orthophosphoric acid to reduce the pH of the digesta resulting in the more concentration of the undissociated form of the acids (Dibner and Buttin 2002). Organic acid reduces inside pH of the bacteria after dissociation and results in death. Organic acids reduce the total microbial load and consequently reduce the subclinical infection leading to improved digestibility and lessened demand of energy by the gut-associated tissue (Khan et al. 2016a). In addition, the low pH of the upper GIT also favors the antimicrobial activity of the organic acids and helps their absorption through diffusion in the epithelium (Scicutella et al. 2021). In addition, the organic acid in the lower part of the intestines reduces the competition of the host with the microflora resulting in improved digestion (Khan and Iqbal 2016).

## Nutrient digestibility

In a study, Garcia et al. (2007) reported that supplemented with 5000 and 10,000 ppm formic acid produced similar results to avilamycin, which ultimately improved the growth and apparent ileal digestibility. In addition, positive effect on the intestinal mucosa was also observed. Cakir et al. (2008) reported that organic acid supplementation significantly improved duodenal villus height. Chaveerach et al. (2004) revealed that acidified drinking water improved the gut epithelial cells. Runho et al. (1997) reported improved metabolizable energy in broiler fed with 0.5–1% fumeric acid. Hernandez et al. (2006) reported that the addition of 5 and 10 g/kg formic acid has little effect on the ileal digestibility of nutrients. A number of authors have documented the positive effect of organic acids on the improved digestibility of nutrients in broiler feed (Hernandez et al. 2006; Garcia et al. 2007; Ghazala et al. 2011; Rodjan et al. 2017; Sureshkumar et al. 2021). Organic acid lowers the pH of the digesta and raises the gastric proteolytic activity (Khan et al. 2016b). The pH modulation of the specific area of the intestines is another factor which favors the proliferation of the specific microbial population, affecting the digestion and absorption of the nutrients, since most of the pathogenic bacteria reside at a pH close to 7 (Sureshkumar et al. 2021). In contrast, the beneficial bacteria survive better at a pH 5.8–6.2. Since organic acids lower the pH, so the acidic environment favors the growth of the beneficial bacteria and reduces the population of the pathogenic microbes (Haque et al. 2009). Ultimately, the digestion and absorption of the nutrients are increased in the presence of the beneficial bacteria. The organic acids probably also activate the pepsin activity which causes proteolysis of protein. As a result, the protein contents are broken down into simple peptides which stimulate the release of gastrin and cholecystokinin hormones. In addition, the pancreatic juice is secreted in an organic acid environment containing higher concentrations of procarboxy peptidases, chymotrypsinogen, and trypsin (Adil et al. 2010). The slower rate of the passage of digesta in response to organic acid enhances the absorption of the feed contents from the intestines (Abudabos et al. 2017). In the presence of acidic environment, bacterial metabolites such as ammonia and amines are reduced (Samanta et al. 2010) which enhances the digestibility. The enhanced nutrient digestibility by organic acid is also associated with improvement in the release of digestive enzymes, microbial phytase activity, and increased pancreatic activity in the gut (Hernandez et al. 2006). In the presence of dietary organic acid, the digestibility of minerals, particularly phosphorous and calcium, has been increased probably due to the more effective role of the *Lactobacillus* spp*.* in the digestive tract. Chowdhury et al. (2009) reported that phosphorous utilization was increased in response to citric acid supplementation in broilers. Similar results were also reported by some other researchers (Nezhad et al. 2007). Dietary supplementation with mixed organic acid increased the pancreatic activity and boosted the expression of tight junction proteins, resulting in healthier broiler production (Ma et al. 2021). Sorbic acid and fumaric acid were also reported to improve significantly trypsin, lipase, and chymotrypsin activities in the intestine at different stages of growth in broilers (Yang et al. 2018).

Birds are well recognized for their inability to efficiently metabolize fibrous carbohydrates in their diet. Soluble dietary fiber increases digesta viscosity and slows passage rate, ultimately, lowering the absorption of nutrients (Shang et al. 2020). The gut microbiota is crucial in the fermentation of undigested carbohydrates, especially in the caeca. This fermentation produces acetate, propionate, and butyrate, all of which have been shown to enhance avian intestinal morphology, tight junctions, and immunological state Corrêa-Oliveira et al. (2016). The usage of organic acids has also been linked to improvements in nutritional digestibility, particularly for minerals (Emami et al. 2013).

## Immune response

In modern poultry production, broilers appear to have less resistant immune system. The broilers are expected to produce a high meat yield within a short period of time. The fast growth and efficient feed utilization have resulted in compromised immune status in broiler (Khan et al. 2012a; Ghazvinian et al. 2018). In broilers, it is assumed that there is a close relationship between the immune status and their genetic makeup, resulting in higher susceptibility of compromised immune response compared to the other species of birds (Emami et al. 2013). Since the growth rate in broilers is rapid, however, their immunity level is not developed at the same speed; therefore, they are susceptible to many infectious diseases. This response is more weakened in the absence of antibiotics in broiler feed. Organic acids are capable of modulating the number of pathogenic bacteria and therefore, they might be helpful in improving the immune status of broiler. An improved immune response was reported in broiler chicks fed with different blends of organic acids (Chowdhury et al. 2009; Houshmand et al. 2012; Abbas et al. 2013; Yang et al.^2018^; Scicutella et al. 2021).

A significant increase in antibody titer was significantly improved in broilers against Newcastle disease in response to organic acid supplementation (Houshmand et al. 2012; Abbas et al. 2013). The improved immunoglobulin status was also found to be raised by the dietary supplementation of organic acid in broilers (Park et al. 2009). Heavier immune organs have also been reported in response of organic acid supplementation (Ghazala et al. 2011). Similarly, improved immune response has also been reported in response to organic acid supplementation in broiler in other studies. The improved immune response of broiler could be due to the increased *Lactobacillus* spp*.* population in the gastrointestinal tract, which have a positive effect on the host immune system (Emami et al. 2013). The enhanced immune response of broiler in the presence of sodium butyrate has been attributed to its ability to inhibit NF-kB activation (Zhang et al. 2011). Also, Zhang et al. (2011) reported increased antioxidant activity and reduced oxidant status in broiler fed with sodium butyrate. Different doses of organic acid (0.2% and 0.4%) blends increased IgG titer (primary and secondary) and humoral immunity to a dose of sheep RBCs and vaccination of bursal disease (IBD) virus and infectious bronchitis (Emami et al. 2013; Emami et al. 2017). Dietary supplementation with mixed organic acid improved immunological features in blood and the small intestine, as well as modulated the cecum bacterial population, resulting in healthier broiler development (Ma et al. 2021). Haque et al. (2009) suggested that the citric acid appears to stimulate the nonspecific immunity since it increased lymphocyte number in the lymphoid organs. The methods by which organic acids regulate immunity are unknown, although it is speculated that organic acid causes activation of systemic regulatory T cells and a reduction in an inflammatory response signal (alpha 1-acid glycoprotein) in broilers following H9N2 vaccination (Lee et al. 2011). Gut-related immunity in response to organic acid supplementation also depends upon particular GIT bacteria, and their association with gut-associated lymphoid tissue (Emami et al. 2017).

## Intestinal integrity

It has been demonstrated that organic acid-supplemented meals protected epithelial cells against disruption by reducing the synthesis of toxins in the gut, resulting in reduced mucosal permeability of the intestines (Kumar et al. 2021). Good intestinal health is the primary requirement in the poultry industry to achieve target feed efficiency and growth rate. The antimicrobial agents reduce the pathogen load and their toxins in the intestines. Short chain fatty acids stimulate healthy tissue turnover and enhance the proliferation of normal crypt cells (Scheppach et al. 1995). Organic acids in different doses and combinations have shown improved villus height, width, crypt depth, and area of ileum, duodenum, and jejunum in broiler (Leeson et al. 2005; Garcia et al. 2007; Panda et al. 2009; Adil et al. 2010; Rodjan et al. 2017; Yang et al. 2018). Recently, Kumar et al. (2021) reported that short chain fatty acids consisting of different blends of organic acid improved the intestinal health in broilers infected with necrotic enteritis. Organic acids stimulate the proliferation of gastrointestinal cells by increasing ileal proglucagon mRNA glucagon-like peptide-2 (GLP-2) and protein expression which can mediate epithelial proliferation of the intestines.

The most acceptable reason of the improved intestinal integrity has been associated with a reduction of the intestinal pathogenic bacteria. The pathogenic bacteria alter the normal microflora and disturb the permeability of the intestines, and thereby facilitate the entry of toxins into the intestines leading to the inflammatory process. Consequently, there is a reduction in the texture of the villus, which ultimately decreases the absorptive capacity of the intestines. The organic acids reduce the number of pathogenic bacteria and their entry into the intestinal mucosa and subsequently the inflammatory process (Hayat et al. 2014; Sultan et al. 2015; Abudabos et al. 2016; Khan et al. 2016b). Some studies also showed no effect on the intestinal health in the diets supplemented with organic acids (Milbradt et al. 2014; Waguespack Levy et al. 2015). Disparity across studies is definitely due to changes in the underlying microbiological challenge and bird performance, as well as discrepancies in organic acids employed, dosage, feed formulations, and other factors that must be taken into account for the optimal field responses.

## Conclusion

Poultry producers are experiencing huge challenges of increasing demand of production and health. It is a well-known scientific fact that organic acid is a valuable source of potentiating the growth and prevention of some of the infectious diseases. Organic acids could be a good alternative to antibiotics. Organic acids provide a sustainable and potent alternative to maximize future production and health of broiler. The tremendous potential of organic acids could be advanced with the use of modern science and technologies at molecular, biotechnological, and nanotechnological level. The future studies should be directed towards the optimization of the dose, duration, concentration, and exact mechanism of action of organic acids to authenticate and widen the beneficial uses.

## Data Availability

Not applicable.
